# Current Trends in Intentional Replantation Treatment Among Endodontists and Postgraduate Students in India, the United States of America, and the United Kingdom: A Cross-Sectional Study

**DOI:** 10.7759/cureus.39742

**Published:** 2023-05-30

**Authors:** Vijayaragavan Praveen Kumar, Kadandale Sadasiva, Jwaalaa Raj kumar, Anupama Ramachandran, Revathy Parthasarathy, Yashini Thanikachalam

**Affiliations:** 1 Conservative Dentistry and Endodontics, Chettinad Dental College and Research Institute, Chennai, IND; 2 Conservative Dentistry and Endodontics, Sree Balaji Dental College and Hospital, Chennai, IND

**Keywords:** intentional replantation, tooth replantation, prognostic indicator, retrograde root-end filling, endodontic microsurgery, survival rate, global survey

## Abstract

Objective

This study aimed to investigate the knowledge, attitude, and practices (KAP) of intentional replantation among postgraduate students and endodontists in India, the United States of America, and the United Kingdom.

Materials and methods

The sample size was estimated using G*Power. Based on the pilot study done before with 60 participants, a sample size of 928 was obtained. The survey consisted of 22 questions, which were finalized after content validation by two endodontic experts. It was circulated through multiple online social platforms such as Instagram, Facebook, WhatsApp, and other online dental communities/channels. The respondents were questioned about the case selection, extraction methods, antibiotic therapy, patient acceptance level, operator preference, prognostic indicator, and various other steps in the intentional replantation treatment modality. The data for this KAP survey were organized in an Excel sheet, and statistical analysis was done using the Chi-squared test. Analysis of descriptive and inferential statistics was conducted using SPSS version 20.0 (IBM Inc., Armonk, New York). A p-value of <0.05 was considered significant.

Results

A strong statistical difference was found in the KAP of the practitioners in different countries. The vast majority (72.7%) considered intentional replantation as an adjunct treatment modality rather than a last resort. A total of 76.5% of the respondents preferred replantation of the tooth into the socket within 15 minutes, and 86.4% of the participants regarded replantation as the most cost-effective treatment modality. Ultrasonics (76.8%) was most commonly chosen for retrograde preparation, and Biodentine (60.1%; Septodont, Saint-Maur-des-Fossés, France) as root-end filling material.

Conclusion

It can be concluded that a broad majority of practitioners in different countries view intentional replantation as an adjunct treatment modality rather than a last resort. Thus, intentional replantation seems to be a promising option for preserving the natural dentition of teeth with high survival rates and better outcomes.

## Introduction

The primary purpose of endodontic treatment is to cure pulpal and periapical pathosis [1]. For dental healthcare practitioners, especially endodontic experts, an inflammatory apical or peri-radicular lesion in an already obturated tooth that persists or progresses is a serious concern. According to different studies [2, 3], the prevalence of this post-treatment endodontic disease varies from 16 to 65%.

The presence of microorganisms throughout the root canal system and/or in the apical region has been suggested as the major reason for pulpal and periapical pathosis, while other factors such as the presence of cysts, cystic components such as cholesterol crystals, and persistent inflammatory activities were also seen to be associated [2]. Nonsurgical retreatment or surgical management is always the primary line of treatment [3].

Intentional replantation can be defined as the purposeful extraction of a tooth from its socket, followed by evaluation of the root surfaces, apicoectomy, retrograde preparation and restoration, and re-insertion of the tooth into its native socket [4]. Intentional replantation is advocated as an alternative way for treating post-endodontic pathology or failed nonsurgical retreatment. It is also useful in cases where the surgical approach to the site is difficult, and there is a need to avoid damaging the innate anatomical structures or to improve operational efficacy [5].

Techniques of intentional replantation based on modern endodontic surgical principles and the understanding of post-replantation complications have continuously evolved and critically advanced [6]. However, despite the improved outcomes based on modern endodontic surgical principles, the knowledge and use of intentional replantation as a treatment option in daily practice are not widespread, possibly because of a shortage of extant literature and a lack of understanding regarding its diverse steps and practices observed in different countries. To the best of our knowledge, there have been no surveys on intentional replantation on a multinational scale. Hence, the aim and purpose of this study were to investigate the knowledge, attitude, and practices (KAP) of intentional replantation as a treatment modality among endodontists and postgraduate students of endodontics in India, the United States of America (USA), and the United Kingdom (UK).

## Materials and methods

This cross-sectional study was carried out from March 2022 to September 2022 and involved endodontists and postgraduate students of endodontics across the globe. The Institutional Human Ethics Committee of Chettinad Academy of Research and Education, registered with the Central Drugs Standard Control Organization, approved the study protocol (140/IHEC/Jan 2022). The sample size was estimated using G*Power. Based on the pilot study done prior with 60 participants, the sample size was calculated, keeping the effect size at 0.5, the alpha error at 0.05, and the power (1-β err prob) at -0.95. The final sample size obtained was 928, which includes 468 postgraduate students of endodontics and 460 endodontists.

Initially, a set of 28 questions were drafted, of which 22 questions were finalized for the survey after content validation (by one national and one international endodontic expert). The questionnaire was developed using Google Forms (Google Inc., Mountain View, California). The survey was circulated through multiple online social platforms such as Instagram, Facebook, WhatsApp, and other online dental communities or channels to postgraduate students of endodontics and endodontists in India, the USA, and the UK.

The study participants were questioned about the selection of the case, extraction methods, antibiotic therapy, root hydration medium, retrograde preparations, retrograde restorative materials, operator preference, prognostic indicators, and patient acceptance level. The reliability was checked by randomly asking the participants to fill out the survey one more time after 15 days.

Statistical analysis

The data for this knowledge, attitude, and practices (KAP) survey were stored in an Excel spreadsheet (Microsoft, Redmond, Washington). Reliability scores for the survey were calculated using the kappa test. Quantitative variables were summarized using frequency and percentage. Descriptive and inferential statistics were analyzed by SPSS Statistics for Windows, version 20.0 (IBM Inc., Armonk, New York). Statistical analysis was done using the Chi-squared test. A p-value of <0.05 was considered significant.

## Results

**Figure 1:**
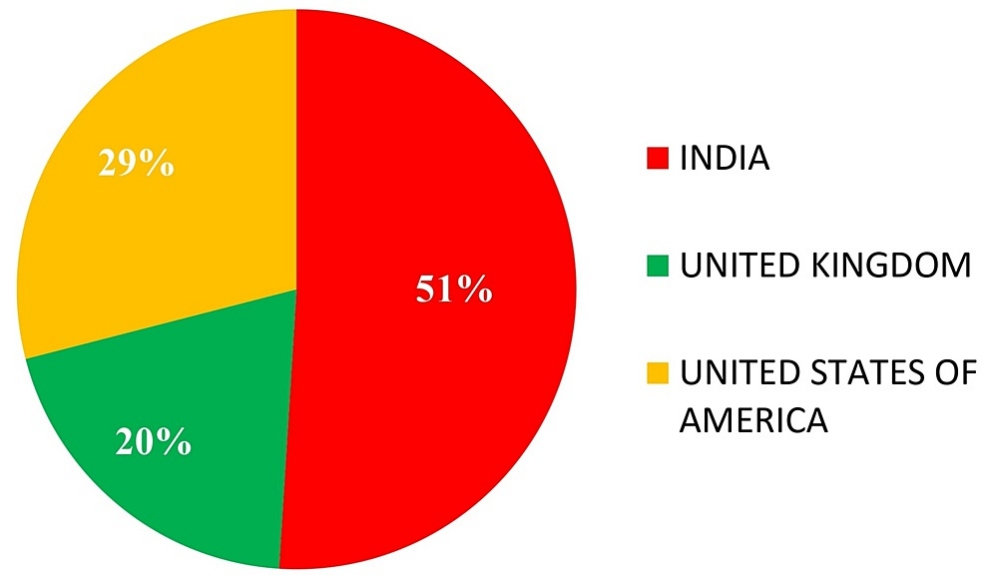
Demographic details of the participants

The questionnaire was circulated to 928 participants, but only 893 responses were achieved, denoting a response rate of 96.2%. Among the respondents, 48.6% were endodontists, and 51.4% were postgraduate students of endodontics. Further demographic details are given in Figure 1.

The reliability of the respondents, calculated using kappa scores, was 0.987. In this study, the Chi-squared test yielded a highly statistically significant (p-value <0.05) association between country and variables on the questionnaire. Regarding the 22 questions of the survey, statistically significant differences were found between the groups and the region of practice.

Regarding the question on participants' attitudes toward intentional replantation, 72.7% considered it as another treatment modality, while 27.3% considered it as a last resort. The participants were aware of the case selection, absolute indications, and contraindications of the treatment modality. Moreover, 86.4% of the participants considered intentional replantation to be more economical than tooth replacement by single-tooth implants. Only 24.6% of the participants were knowledgeable about the survival rate of the intentionally replanted teeth. Most of the respondents were correct about the judicious usage of antibiotics, and 77.3% of the population preferred two operators to perform the treatment.

Pre-operative disinfection of the surgical site was recommended by 92.6% of the participants. Forceps were found to be preferred for extraction by 58.5% of the participants. When asked about the critical step, most of the respondents selected preservation of the socket, periodontal ligament, and alveolar bone. Hanks' Balanced Salt Solution (HBSS) was found to be the most preferred root hydration medium for the storage of the tooth after extraction. A total of 76.5% of the respondents preferred that the tooth be replanted into the socket in less than 15 minutes. Additionally, 78.1% of the respondents preferred to re-insert the tooth with digital placement and digital compression of the socket walls.

**Figure 2:**
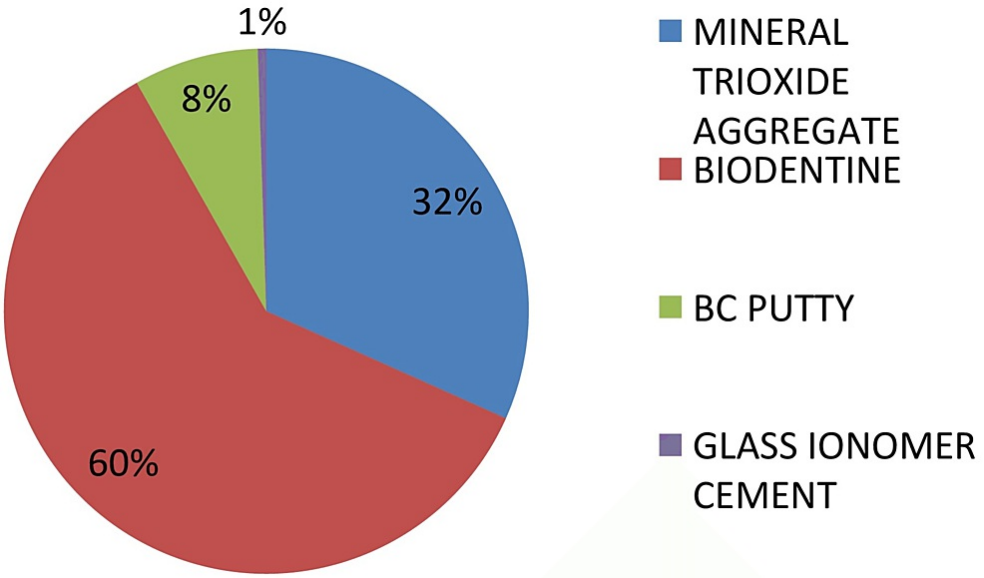
Choice of root-end filling material

Most of the participants preferred ultrasonics for retrograde preparation, and Biodentine (Septodont, Saint-Maur-des-Fossés, France) was the most frequently used material to fill root ends, followed by mineral trioxide aggregate (MTA), as can be seen in Figure 2.

When asked about the intentional replantation of periodontally involved teeth, 68.2% of the participants considered it as an absolute contraindication. Regarding the definitive prognostic indicator for improved clinical outcomes and survival rate, 59% of the respondents preferred teeth with less extra-oral time and pre-operative orthodontic extrusion, while 36.1% chose a lesser amount of extra-oral time as the only prognostic indicator.

**Figure 3:**
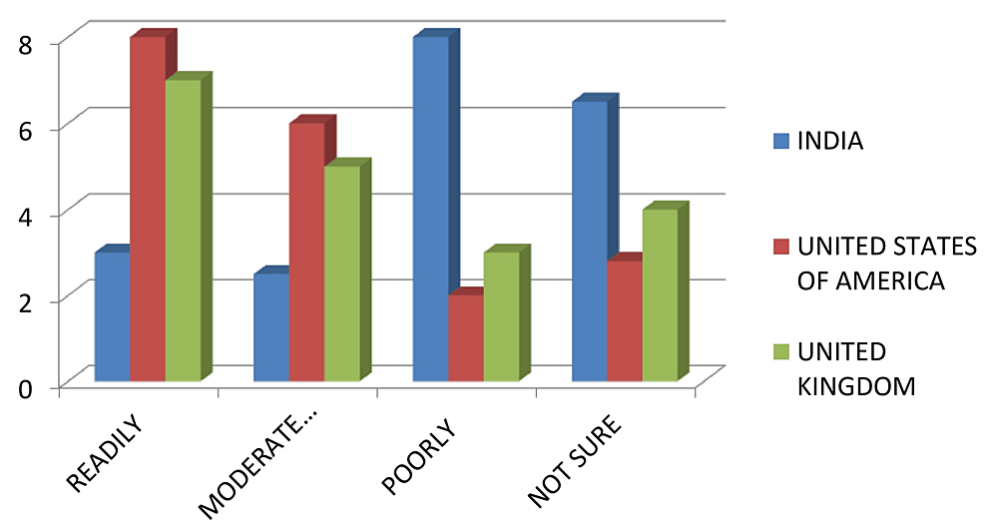
Patient acceptance level of each country

Of the total, 45.4% of the respondents had performed an intentional replantation, out of which only 58.3% had a follow-up of three to five years. When asked about the patient acceptance level toward the treatment modality, 47.1% of the respondents said it was moderately acceptable, 29.9% said it was poorly acceptable, 17% said it was readily acceptable, and 6% said they were not sure about it. The USA and the UK were found to have a better patient acceptance level than India (Figure 3).

## Discussion

To the best of our knowledge, no cross-sectional studies have been conducted on intentional replantation among endodontists. The primary goal of this study was to investigate the KAP of intentional replantation among endodontists and postgraduate students in India, the USA, and the UK and to bridge the gaps that exist in the steps and the protocol of the treatment modality that is observed across different countries.

The participants were aware of the case selection, absolute indications, and contraindications of intentional replantation. However, there was a lack of knowledge among dentists regarding the intentional replantation of periodontally involved teeth. According to Cho et al. [7], intentional replantation is not always contraindicated by periodontal involvement. Moreover, as per a previous study [7], individuals under the age of 40 and those that had teeth with one or two preoperative periodontal pockets less than 6 mm had, respectively, 2.6 and 2.5 times lower probability of failure than older individuals and those that had teeth with higher periodontal pockets.

In this study, intentional replantation was found to be more economical than tooth replacement by implant procedures. This finding agrees with Torabinejad et al. [8]. The participants were well aware of the antibiotic coverage and preoperative disinfection of the surgical site. Most of them preferred two operators to perform the treatment. However, the participants were unaware of the survival rate of intentionally replanted teeth. According to Mainkar et al. [1], the survival rate is 89.1% and may even increase when teeth are replanted based on modern endodontic surgical principles. Regarding atraumatic extraction, the majority of the Indian respondents preferred forceps; on the other hand, dentists from the USA and UK preferred periotome.

HBSS was the largely preferred root hydration medium. The majority (76.8%) preferred ultrasonics for retrograde preparation. This finding agrees with a previous study in which ultrasonics was mostly used for root-end preparation since it prevents micro-crack propagation in root ends, especially in cases of thin roots [9]. In this study, Biodentine was the most commonly selected retrograde filling material, followed by MTA. MTA was favored by most of the Indian dentists.

Regarding prognostic factors for improved clinical outcomes and survival rate, the majority of dentists were only aware of lesser extra-oral time. Very few of them, especially those from the USA and the UK, knew that teeth with preoperative orthodontic extrusion have a better survival rate. According to Cho et al. [9], healing of the intentionally replanted teeth occurs 1.7 times more faster when replanted within 15 minutes (optimal extra oral time - 12.5 minutes), showcasing lesser extra-oral time as a prognostic indicator. According to Choi et al. [10], preoperative orthodontic extrusion about a period of two to three weeks prior to extraction prevents extraction failure as well as post-operative complications and favors positive clinical outcomes. It decreases the risk of resorption and increases the survival rate of the replanted teeth.

When participants were asked about whether they had performed a case of intentional replantation, about 45.4% said they had, with the majority from the USA, followed by the UK and then India. Among those, only 58.3% had a follow-up of three to five years. The USA and the UK were found to have a better patient acceptance level than India. According to Torabinejad et al. [8], there is a reportedly high survival rate (88%) of intentionally replanted teeth. It increases further when the replantation is based on modern endodontic surgical principles; it is higher than the reported healing of nonsurgical endodontic retreatment and also comparable to implant-supported single crowns [11-13]. Intentional replantation can be a suitable option for the management of inaccessible cervical resorptions and vertical root fractures [14].

Due to geographical differences in how English is spoken and understood around the world and the fact that this questionnaire was created to cover the multinational dental population, there is a minor chance that the dentists who responded to it might have unintentionally experienced questionnaire bias. To avoid this form of bias, during the implementation of the pilot study itself, the questions were validated and made as fair and straightforward as possible.

## Conclusions

When nonsurgical and surgical retreatments are considered to be infeasible, intentional replantation emerges as a promising treatment option for preserving the natural dentition. In the present study, responses were gathered on a multinational scale, and the high knowledge scores are encouraging, particularly for specialty dentists. It can be concluded that a vast majority (72.7%) of endodontists and postgraduate students across different countries view intentional replantation as an adjunct treatment modality rather than a last resort. Moreover, it proves to be the most cost-effective alternative compared to single-tooth replacements, with high survival rates and better outcomes.

## Figures and Tables

**Figure 4:**
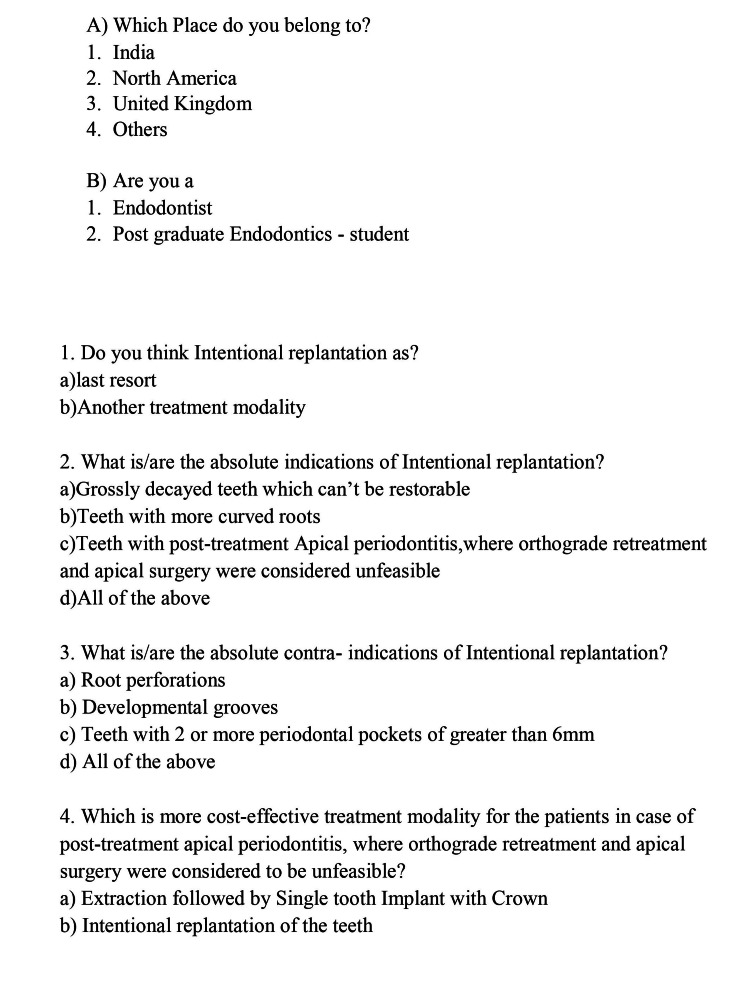
Questionnaire, demographics and questions 1-4

**Figure 5:**
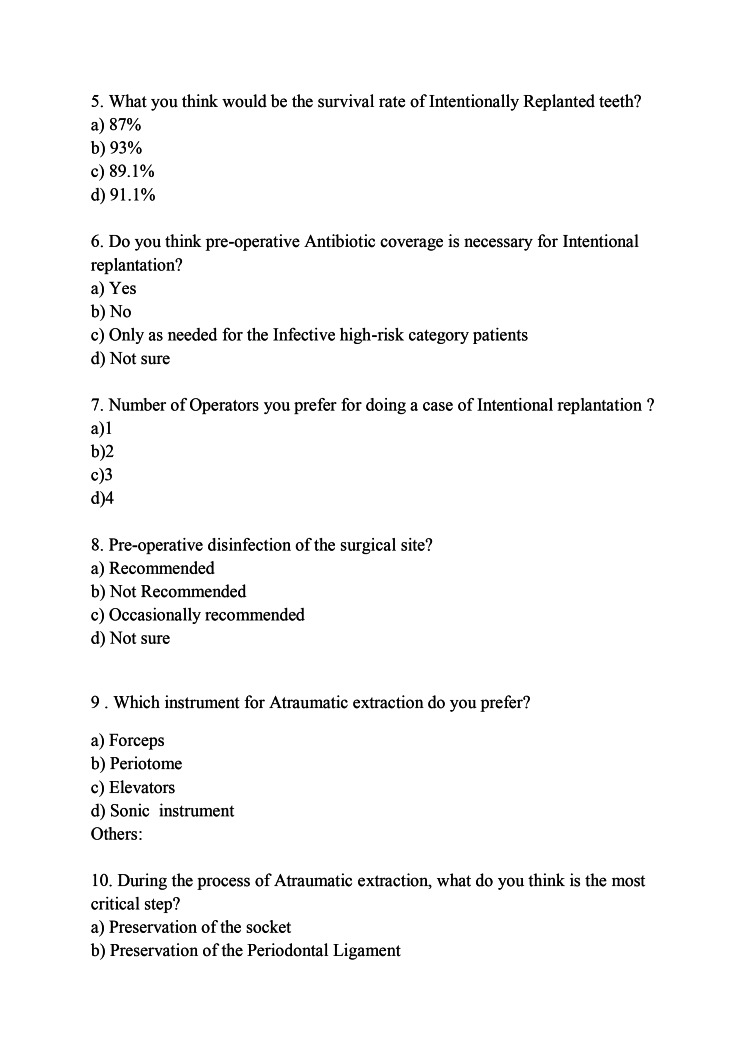
Questionnaire, questions 5-10

**Figure 6:**
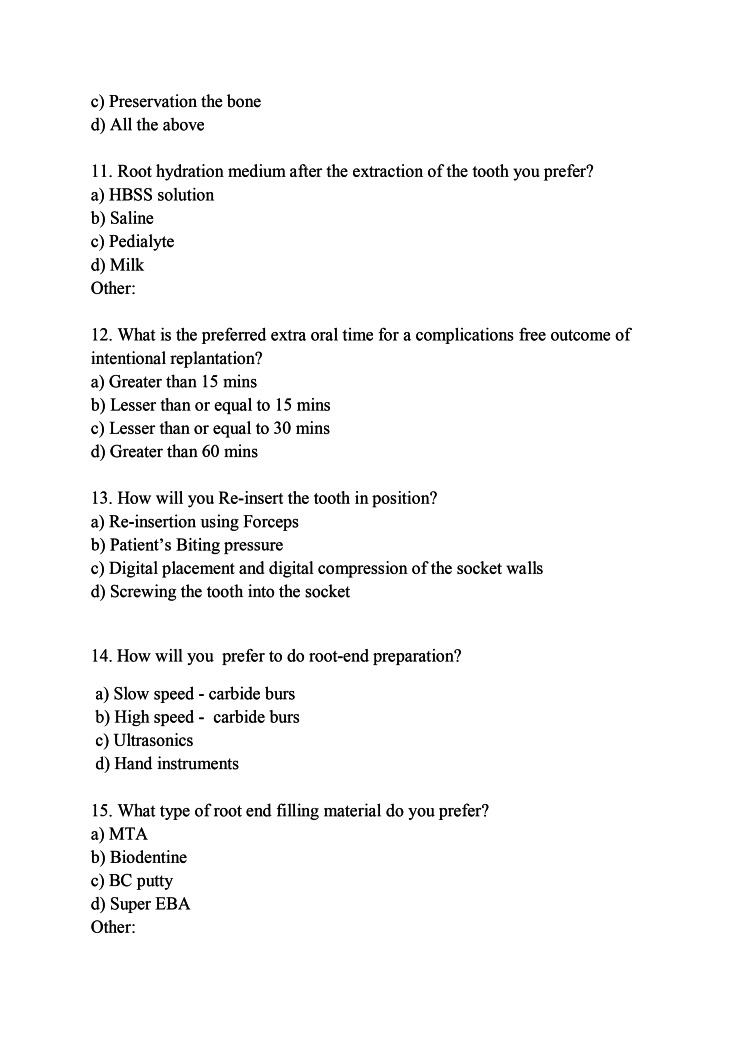
Questionnaire, questions 11-15

**Figure 7:**
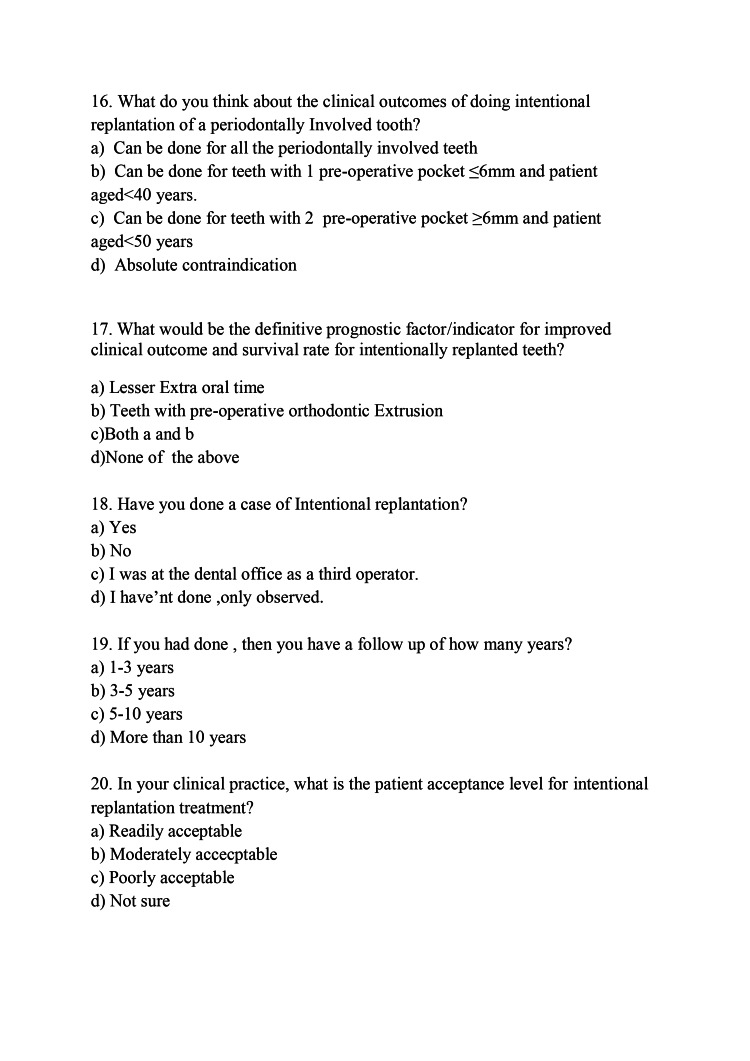
Questionnaire, questions 16-20
